# Maternal iron status during pregnancy and respiratory and atopic outcomes in the offspring: a Mendelian randomisation study

**DOI:** 10.1136/bmjresp-2018-000275

**Published:** 2018-03-30

**Authors:** Annabelle Bédard, Sarah J Lewis, Stephen Burgess, A John Henderson, Seif O Shaheen

**Affiliations:** 1 Centre for Primary Care and Public Health, Barts and The London School of Medicine and Dentistry, Queen Mary University of London, London, UK; 2 Population Health Sciences, Bristol Medical School, University of Bristol, Bristol, UK; 3 MRC Biostatistics Unit, University of Cambridge, Cambridge, UK; 4 Cardiovascular Epidemiology Unit, University of Cambridge, Cambridge, UK

**Keywords:** ALSPAC, iron, pregnancy, lung function, Mendelian randomisation

## Abstract

**Introduction:**

Limited evidence from birth cohort studies suggests that lower prenatal iron status may be a risk factor for childhood respiratory and atopic outcomes, but these observational findings may be confounded. Mendelian randomisation (MR) can potentially provide unconfounded estimates of causal effects by using common genetic variants as instrumental variables. We aimed to study the relationship between prenatal iron status and respiratory and atopic outcomes in the offspring using MR.

**Methods:**

In the Avon Longitudinal Study of Parents and Children birth cohort, we constructed four maternal genotypic risk scores by summing the total number of risk alleles (associated with lower iron status) across single nucleotide polymorphisms known to be associated with at least one of four iron biomarkers (serum iron, ferritin, transferrin and transferrin saturation). We used MR to study their associations with respiratory and atopic outcomes in children aged 7–9 years (n=6002).

**Results:**

When analyses were restricted to mothers without iron supplementation during late pregnancy, negative associations were found between the maternal transferrin saturation score and childhood forced expiratory volume in 1 s and forced vital capacity (difference in age, height and gender-adjusted SD units per SD increase in genotypic score: −0.05 (−0.09, −0.01) p=0.03, and −0.04 (−0.08, 0.00) p=0.04, respectively).

**Conclusion:**

Using MR we have found weak evidence suggesting that low maternal iron status during pregnancy may cause impaired childhood lung function.

**Key messages**

- In the literature there have been clues on the role of maternal iron status and anaemia during pregnancy in the development of respiratory and atopic disorders during childhood. A concern with all observational studies, and particularly in nutritional epidemiology, is that findings may be influenced by confounding. In the present study, we used Mendelian randomisation, which is a form of instrumental variable analysis where a genetic variant can be used as a proxy for a specific exposure, to test whether maternal iron status during pregnancy is likely to be causally related to respiratory and atopic outcomes in the offspring.
- We have found weak evidence suggesting that low maternal iron status during pregnancy may cause impaired childhood lung function.
- Given that iron deficiency is common in pregnancy in the West, these findings might have public health implications and need to be further investigated.

## Background

Epidemiological studies have suggested that maternal nutrition during pregnancy may influence the development of childhood respiratory and atopic disorders,^1 2^ though evidence is conflicting and data are lacking on associations with outcomes beyond 5 years of age. In the West, iron deficiency is common in pregnancy,^3^ but few studies have investigated the role of this potentially modifiable risk factor in the development of asthma and allergies. Prenatal iron deficiency could plausibly influence respiratory and allergic outcomes by causing prematurity and impaired fetal growth. Gestational age at delivery and offspring birth weight are associated with maternal haemoglobin concentration^4^ and with childhood wheezing, asthma, eczema, allergic sensitisation and impaired lung function.^5–8^

In the Avon Longitudinal Study of Parents and Children (ALSPAC), we reported an inverse association between umbilical cord iron concentration and wheezing and eczema in early childhood.^9^ Exploratory analyses in a small subgroup of another UK birth cohort—the SEATON study—suggested associations between different indicators of lower maternal iron status in early gestation and an increased risk of wheezing and lower lung function in the offspring at 10 years of age, although findings were inconsistent across the different indicators, and no associations were found with asthma or eczema.^10^ Iron deficiency is the most common cause of anaemia in pregnancy,^11^ and recent analyses conducted in the ALSPAC birth cohort showed associations between lower maternal haemoglobin concentrations in pregnancy, and an increased risk of atopy, elevated IgE and reduced lung function in the offspring at 7 years of age.^12^ In the USA, a prospective study found positive associations for maternal anaemia with both early childhood wheezing and persistent wheezing,^13^ and a cross-sectional study in children reported positive associations between anaemia and atopic disease.^14^ On the other hand, a prospective study conducted in the Netherlands did not confirm any association between maternal haemoglobin concentrations during pregnancy and wheezing in early childhood or asthma outcomes at the age of 6 years.^15^

A concern with all observational studies, and particularly in nutritional epidemiology, is that findings may be influenced by confounding. Mendelian randomisation (MR) is a form of instrumental variable (IV) analysis where a genetic variant can be used as a proxy for a specific exposure to test whether that exposure is likely to be causally related to an outcome.^16^ A key principle underpinning MR is that genotype is randomly allocated during meiosis, and consequently associations between genetic variants and disease are not generally susceptible to confounding by lifestyle factors. This approach was recently used in ALSPAC to determine whether maternal iron status in pregnancy was causally related to child’s IQ.^17^ The aim of this study was to use MR to determine whether maternal iron status during pregnancy is causally associated with respiratory and atopic outcomes in the offspring in the large population-based ALSPAC birth cohort.

## Methods

### Participants

The ALSPAC is a population-based birth cohort that recruited 14 541 predominantly white pregnant women resident in Avon, UK, with expected dates of delivery from 1 April 1991 to 31 December 1992. These pregnancies resulted in 13 613 singletons who were alive at 1 year of age. The cohort has been followed since birth with annual questionnaires and, since age 7 years, with objective measures in annual research clinics. The study protocol has been described previously^18 19^ and further information can be found at http://www.alspac.bris.ac.uk, which contains details of all the data that are available: http://www.bris.ac.uk/alspac/researchers/data-access/data-dictionary/.

### Outcome assessment

When the children were 7.5 years old, mothers were asked: ‘Has your child had any of the following in the past 12 months: wheezing with whistling; asthma; eczema; hay fever?’ Children were defined as having current doctor-diagnosed asthma at 7.5 years (primary outcome) if mothers responded positively to the question, ‘Has a doctor *ever* actually *said* that your study child has asthma?’ and positively to one or both of the questions on wheezing and asthma in the past 12 months. Atopy at 7 years was defined by skin prick test as a positive reaction (maximum diameter of any detectable weal) to *Dermatophagoides pteronyssinus*, cat or grass (after subtracting positive saline reactions from histamine and allergen weals, and excluding children unreactive to 1% histamine). Serum total IgE (kU/L) was measured at 7 years by fluoroimmunoassay using the Pharmacia UNICAP system (Pharmacia and Upjohn Diagnostics, Uppsala, Sweden).

Lung function was measured by spirometry (Vitalograph 2120) at age 8½ years after withholding short-acting bronchodilators for at least 6 hours and long-acting bronchodilators and theophyllines for at least 24 hours. The best of three reproducible flow-volume curves was used to measure forced expiratory volume in 1 s (FEV_1_), forced vital capacity (FVC) and maximal mid-expiratory flow (FEF_25-75_). Lung function measurements were transformed to age, height and gender-adjusted SD units.^20^ The tests adhered to American Thoracic Society (ATS) criteria for standardisation and reproducibility of flow-volume measurement,^21^ with the exception of ATS recommendations for duration of expiration, since many young children cannot sustain exhalation for 6 s to establish FVC.^22^ We therefore used no volume change over >1 s to define the plateau phase of the flow-volume curve as the end-of-test criterion in those unable to blow >6 s.

### Genetic data and weighted genotypic risk score calculation

Maternal DNA was a mixture of samples extracted from blood samples collected during pregnancy and samples extracted from lymphoblastoid cell lines. ALSPAC mothers were genotyped using the Illumina human660W-quad array at Centre National de Génotypage and genotypes were called with Illumina GenomeStudio. PLINK (V.1.07) was used to carry out quality control measures on an initial set of 10 015 subjects and 557 124 directly genotyped single nucleotide polymorphisms (SNP). After applying rigorous quality control measures, genotype data were available for 8196 unrelated mothers (see online supplementary file for further details of genotype quality control and imputation methods). These data have been used in previous genome-wide association (GWA) studies.^23^ For details on child genotyping, see online supplementary file.

10.1136/bmjresp-2018-000275.supp1 Supplementary file 1

We identified 12 SNPs for analysis, recently shown in the largest GWA meta-analysis of iron biomarkers (Genetics of Iron Status (GIS) consortium) to be associated (p<5×10^−8^) with at least one of four iron biomarkers (serum iron, ferritin, transferrin and transferrin saturation)^24^ (see table 1 for further details). Of these SNPs, five were genotyped and seven were imputed; imputation quality score was high (>0.97) for all SNPs. Pairwise linkage disequilibrium between the 12 selected SNPs was checked (single nucleotide polymorphisms annotator, SNiPA^25^). In order to increase statistical power, we constructed a weighted maternal genotypic risk score^26 27^ for each iron biomarker, comprising the number of risk alleles—defined as those associated with lower iron status (hence the higher the score, the greater the risk of lower iron status)—multiplied by the genome-wide association study (GWAS) effect estimates for each SNP-iron biomarker (serum iron, ferritin, transferrin and transferrin saturation) association,^24^ then summed over all SNPs, as summarised in table 1. As serum iron and other iron biomarkers had not been measured in ALSPAC mothers in pregnancy, we used maternal haemoglobin concentrations in pregnancy as proxy measures of maternal iron status in order to partially validate our IVs in the ALSPAC pregnant women.

**Table 1:** SNPs associated with iron and iron biomarkers in a published genome-wide association meta-analysis^24^

SNPs	Risk allele*	Nearest gene(s)	Effect estimates reported†			
			Iron	Ferritin	Transferrin	Transferrin saturation
rs1799945‡	C	*HFE*	−0.189	−0.065	0.114	−0.231
rs1800562§	G	*HFE*	−0.328	−0.204	0.479	−0.577
rs855791§	A	*TMPRSS6*	−0.181	−0.055	0.044	−0.190
rs8177240†	T/G¶	*TF*	−0.066		0.380	−0.100
rs7385804‡	C	*TFR2*	−0.064			−0.054
rs744653§	T	*WDR75*–*SLC40A1*		−0.089	0.068	
rs651007‡	T	*ABO*		−0.050		
rs411988‡	A	*TEX14*		−0.044		
rs9990333§	C	*TFRC*			0.051	
rs4921915‡	A	*NAT2*			0.079	
rs6486121§	C	*ARNTL*			0.046	
rs174577‡	A	*FADS2*			0.062	

*Associated with lower iron status, lower ferritin status, higher transferrin status or lower transferrin saturation status with p<5.10^−8^.

†From meta-analysis of covariate-adjusted standardised regression coefficients of phenotypic values on the allele count for the risk allele.

‡Imputed SNP.

§Genotyped SNP.

¶T is the risk allele for iron (associated with lower iron status) and G is the risk allele for transferrin and transferrin saturation (associated with higher transferrin status and lower transferrin saturation status).

SNP, single nucleotide polymorphism.

### Maternal and offspring characteristics

Data were available on selected characteristics known (from existing literature) to be associated with one or more of the outcomes of interest.^28^ These included maternal age at delivery, sex of child, season of birth, maternal history of atopic diseases (hay fever, asthma, eczema, allergies, or attacks of wheezing with whistling on the chest or attacks of breathlessness in the past 2 years), parity, highest educational qualification, housing tenure, financial difficulties, gestational age at delivery, birth weight, maternal pre-pregnancy body mass index and maternal factors during pregnancy (smoking status, anxiety score, paracetamol use, antibiotic use, infections, total energy intake and use of iron supplementation in early (<18 weeks) and late (20–32 weeks) pregnancy). Smoking status was categorised as the maximum exposure during pregnancy (never, passive smoking only, 1–9 cigarettes per day, 10–19 cigarettes per day, ≥20 cigarettes per day).

### Measurement of haemoglobin

Maternal blood haemoglobin measurements (g/dL) were taken as part of routine antenatal care and abstracted from the women’s obstetric records by six trained research midwives. We derived the first haemoglobin as the haemoglobin measurement with the earliest gestational age for each woman, provided that this was before 18 weeks’ gestation. If there was not a measurement prior to 18 weeks’ gestation, this variable was treated as missing. We derived the last haemoglobin as the haemoglobin measurement with the latest gestational age for each woman, provided that this was after 28 weeks’ gestation, otherwise this variable was set to missing.

### Statistical analysis

Multiple births were excluded from the analyses. Although the ALSPAC population is largely white, mother-child pairs were excluded from all analyses if the mother’s reported ethnicity was non-white or unknown. To address possible residual confounding by population substructure, we controlled for 10 variables derived by principal component analysis (PCA) from ALSPAC GWAS data.^29^ The distributions of maternal and offspring characteristics were compared across maternal genotypic risk score quartiles using F statistics for differences in continuous variables, and Χ^2^ tests for differences in categorical variables. The main analyses were further adjusted for any maternal or offspring characteristic which was found to be associated with maternal genotypic risk scores. Logistic and linear regression was used to analyse associations for individual SNPs (per risk allele effects) and maternal genotypic risk scores (per SD increase effects) with binary and continuous outcomes, respectively. After log-transforming total IgE, linear regression was used to estimate geometric mean ratios for IgE; confidence limits were calculated using Huber variances. Assumptions of Hardy-Weinberg equilibrium were formally tested using a likelihood ratio test and the asymptotic p value is reported.

**Figure 1:**
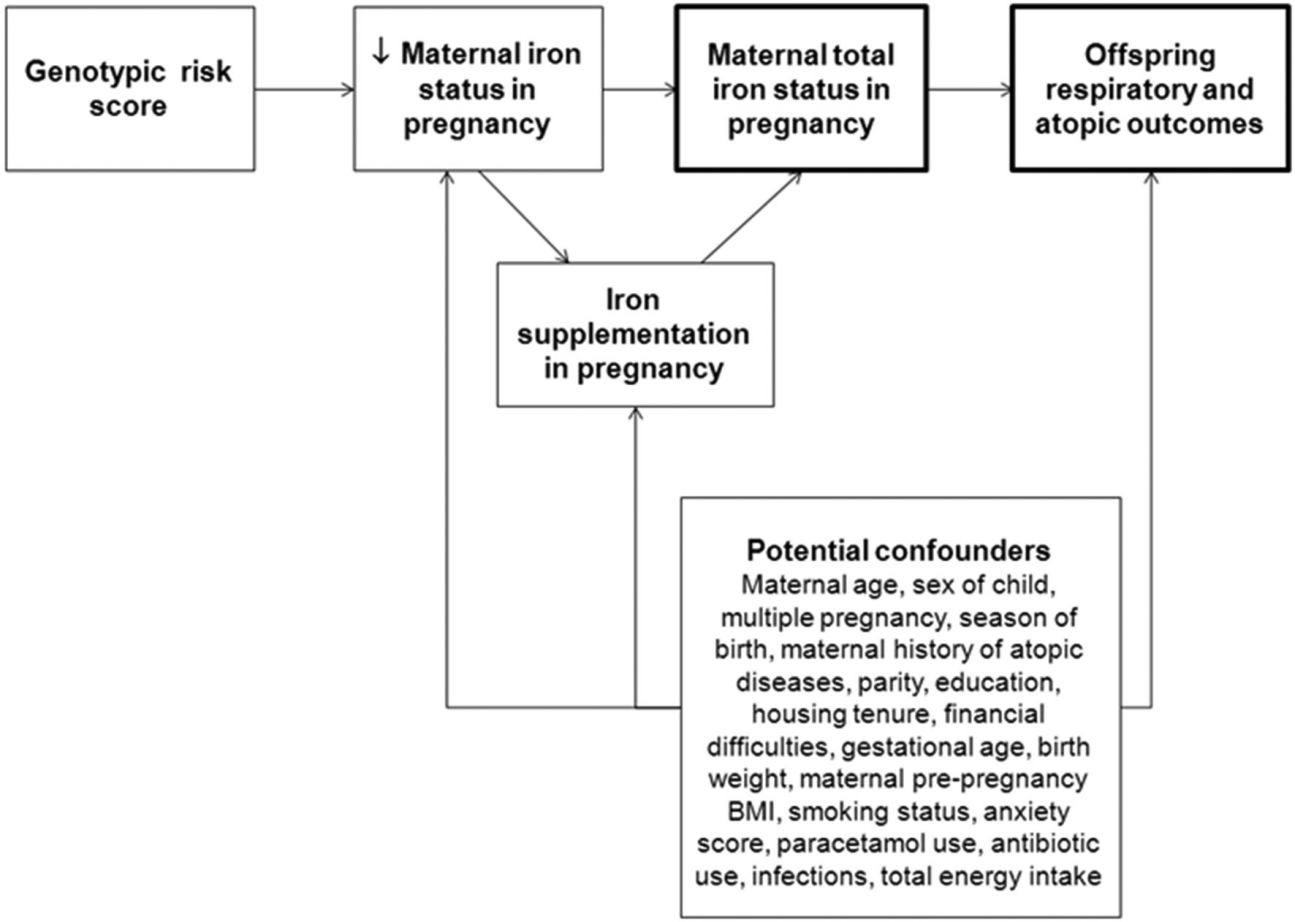
Directed acyclic graph showing potential confounders of the association between maternal total iron status in pregnancy (exposure of interest) and offspring respiratory and atopic outcomes (outcomes of interest) assessed using Mendelian randomisation. BMI, body mass index.

Figure 1 shows a directed acyclic graph to illustrate potential confounders of the associations between maternal iron status in pregnancy and offspring respiratory and atopic outcomes assessed using MR. As many of the ALSPAC women were supplemented with iron because of iron deficiency anaemia, especially in late pregnancy, and this could potentially affect the reliability of the IV as a predictor of maternal iron status, and dilute associations with childhood outcomes (see figure 1), we conducted sensitivity analyses stratifying our study population by iron supplementation in late pregnancy. However, if the probability of an individual choosing to take iron supplements depends on their current iron status, then stratification on supplementation status could introduce collider bias.^30^ This could lead to associations between the genetic score and outcome variables within the strata, even if there is no causal effect of iron status on the outcome. Therefore, a simulation study using parameters derived from the substantive analysis in the paper was conducted to assess the likely type 1 error rate for detecting a causal effect at different degrees of differential supplementation (see online supplementary file for further details). To address the issue of potential overlap between maternal and offspring genetic variants, and assess the extent to which this might have confounded the associations between the maternal genotypic scores and childhood outcomes, we studied the associations between the child’s genotypic scores and childhood outcomes. We also conducted MR-Egger and weighted median sensitivity analyses to assess potential pleiotropy and the likelihood of a causal effect.^31^ All statistical analyses were carried out using Stata V.12.1 (StataCorp LP, USA).

## Results

Of the 13 613 singletons alive at 1 year of age, information on maternal genotype was available for 8079, of whom 681 were excluded because of mother’s reported ethnicity (non-white/unknown). Of the remainder, there was information on at least one of the outcomes of interest for 6002 children (online supplementary figure 1).

No linkage disequilibrium was detected among the 12 SNPs (all pairwise correlations between the 12 variants had an R²<0.10). No strong evidence of Hardy-Weinberg disequilibrium was found among the 12 SNPs (online supplementary table 1). The maternal genotypic iron, ferritin, transferrin and transferrin saturation scores were based on five SNPs, six SNPs, nine SNPs and five SNPs, respectively (see table 1 for list of included SNPs). There was strong evidence (p trend <5.10^−4^) to suggest that three of the SNPs which were included in all four scores (rs1799945—nearest gene: *HFE*, rs1800562—nearest gene: *HFE* and rs855791—nearest gene: *TMPRSS6*), and the four maternal genotypic risk scores, were negatively associated with maternal haemoglobin concentrations in early and late pregnancy (table 2). When we stratified by iron supplementation in late pregnancy, although negative associations were found in both strata between the four genotypic scores and maternal haemoglobin in late pregnancy, stronger associations were mostly observed among unsupplemented women (table 3).

**Table 2:** Associations between maternal genotype at individual iron-related SNPs, maternal genotypic risk scores and haemoglobin concentrations in early and late pregnancy in ALSPAC

SNPs	Risk allele*	Early pregnancy (n=5219)		Late pregnancy (n=5438)	
		β† (95% CI)	P trend	β† (95% CI)	P trend
rs1799945	C	−0.07 (−0.12 to –0.02)	0.005	−0.09 (−0.14 to –0.04)	2.3×10^−4^
rs1800562	G	−0.13 (−0.20 to –0.06)	1.5×10^−4^	−0.19 (−0.26 to –0.13)	1.1×10^−8^
rs855791	A	−0.11 (−0.15 to –0.08)	1.8×10^−10^	−0.12 (−0.15 to –0.08)	1.3×10^−11^
rs8177240	T/G‡	0.02 (−0.02 to 0.05)	0.34	−0.02 (−0.06 to 0.01)	0.23
rs7385804	C	0.01 (−0.03 to 0.04)	0.77	0.00 (−0.03 to 0.04)	0.79
rs744653	T	−0.04 (−0.09 to 0.01)	0.10	0.00 (−0.04 to 0.05)	0.88
rs651007	T	−0.04 (−0.08 to 0.00)	0.07	−0.05 (−0.09 to –0.01)	0.02
rs411988	A	−0.02 (−0.06 to 0.01)	0.17	0.00 (−0.03 to 0.03)	0.99
rs9990333	C	0.01 (−0.03 to 0.04)	0.71	0.01 (−0.03 to 0.04)	0.69
rs4921915	A	−0.03 (−0.07 to 0.01)	0.15	−0.02 (−0.06 to 0.02)	0.43
rs6486121	C	0.00 (−0.03 to 0.04)	0.96	0.01 (−0.03 to 0.04)	0.62
rs174577	A	0.00 (−0.03 to 0.04)	0.79	−0.01 (−0.05 to 0.03)	0.57
Iron score		−0.09 (−0.11 to –0.06)	2.3×10^−12^	−0.11 (−0.13 to –0.08)	1.8×10^−18^
Ferritin score		−0.09 (−0.12 to –0.07)	6.1×10^−13^	−0.09 (−0.12 to –0.07)	8.2×10^−14^
Transferrin score		−0.05 (−0.07 to –0.03)	4.8×10^−5^	−0.04 (−0.07 to –0.02)	7.6×10^−4^
Transferrin saturation score		−0.09 (−0.11 to –0.06)	9.8×10^−13^	−0.11 (−0.12 to –0.08)	5.5×10^−16^

*Associated with lower iron status, lower ferritin status, higher transferrin status or lower transferrin saturation status with p<5.10^−8^.

†Per risk allele effect estimates are reported for SNPs, per SD increase effect estimates are reported for scores.

‡T is the risk allele for iron (associated with lower iron status) and G is the risk allele for transferrin and transferrin saturation (associated with higher transferrin status and lower transferrin saturation status).

ALSPAC, Avon Longitudinal Study of Parents and Children; SNP, single nucleotide polymorphism.

**Table 3:** Associations of maternal genotypes and genotypic risk scores with haemoglobin concentrations in late pregnancy stratified by iron supplementation in late pregnancy

SNPs	Risk allele*	Unsupplemented women (n=3055)		Supplemented women (n=2248)	
		β† (95% CI)	P trend	β† (95% CI)	P trend
rs1799945	C	−0.12 (−0.19 to –0.06)	1.3×10^−4^	−0.04 (−0.12 to 0.04)	0.31
rs1800562	G	−0.22 (−0.30 to –0.13)	7.3×10^−7^	−0.14 (−0.25 to –0.03)	0.01
rs855791	A	−0.09 (−0.14 to –0.05)	8.6×10^−5^	−0.14 (−0.19 to –0.09)	2.0×10^−7^
rs8177240	T/G‡	−0.04 (−0.09 to 0.01)	0.12	−0.01 (−0.06 to 0.05)	0.81
rs7385804	C	0.00 (−0.05 to 0.05)	0.95	0.00 (−0.05 to 0.06)	0.86
rs744653	T	−0.04 (−0.11 to 0.02)	0.18	0.06 (−0.02 to 0.13)	0.14
rs651007	T	−0.05 (−0.11 to 0.01)	0.08	−0.04 (−0.10 to 0.03)	0.27
rs411988	A	−0.02 (−0.06 to 0.03)	0.46	0.02 (−0.04 to 0.07)	0.54
rs9990333	C	0.04 (0.00 to 0.09)	0.07	−0.05 (−0.10 to 0.00)	0.06
rs4921915	A	−0.02 (−0.07 to 0.04)	0.55	0.00 (−0.07 to 0.06)	0.91
rs6486121	C	0.00 (−0.04 to 0.05)	0.86	0.02 (−0.03 to 0.08)	0.40
rs174577	A	−0.01 (−0.05 to 0.04)	0.76	−0.02 (−0.08 to 0.03)	0.41
Iron score		−0.12 (−0.15 to –0.09)	7.5×10^−13^	−0.09 (−0.13 to –0.05)	4.4×10^−6^
Ferritin score		−0.11 (−0.15 to –0.08)	2.0×10^−12^	−0.06 (−0.09 to –0.02)	4.5×10^−3^
Transferrin score		−0.04 (−0.07 to –0.01)	0.01	−0.04 (−0.07 to 0.00)	0.06
Transferrin saturation score		−0.11 (−0.14 to –0.07)	8.1×10^−11^	−0.08 (−0.12 to –0.04)	2.8×10^−5^

*Associated with lower iron status, lower ferritin status, higher transferrin status or lower transferrin saturation status with p<5.10^−8^.

†Per risk allele effect estimates are reported for SNPs, per SD increase effect estimates are reported for scores.

‡T is the risk allele for iron (associated with lower iron status) and G is the risk allele for transferrin and transferrin saturation (associated with higher transferrin status and lower transferrin saturation status).

SNP, single nucleotide polymorphism.

With increasing maternal genotypic iron score (most predictive of low iron status), usage of iron supplements increased, especially during late pregnancy. The iron score was not associated with any other maternal or offspring characteristics (online supplementary table 2). Similar findings were obtained when maternal and offspring characteristics were studied according to the other maternal genotypic scores (data not shown). After controlling for iron supplementation in pregnancy and population substructure, no association was found between the maternal genotypic iron, transferrin and transferrin saturation scores and childhood atopic outcomes (table 4 and online supplementary table 3). Similar findings were obtained when the study population was restricted to unsupplemented mothers (table 5 and online supplementary table 4).

**Table 4:** Associations between maternal genotypic scores and atopy, asthma, FEV_1_, FVC and FEF_25-75_ in the offspring

	OR or β*† (95% CI)			
	Iron score	Ferritin score	Transferrin score	Transferrin saturation score
Atopy (n=3943)				
Per SD increase	1.02 (0.95 to 1.10)	1.00 (0.92 to 1.08)	0.97 (0.90 to 1.04)	1.00 (0.93 to 1.08)
P for trend	0.60	0.93	0.38	0.96
Asthma (n=4873)				
Per SD increase	1.05 (0.96 to 1.14)	0.97 (0.89 to 1.06)	0.97 (0.89 to 1.05)	1.03 (0.94 to 1.12)
P for trend	0.31	0.54	0.47	0.56
FEV_1_ (n=4014)				
Per SD increase	−0.02 (−0.05 to 0.01)	−0.01 (−0.04 to 0.02)	−0.01 (−0.05 to 0.02)	−0.02 (−0.05 to 0.01)
P for trend	0.26	0.48	0.34	0.14
FVC (n=4086)				
Per SD increase	0.00 (−0.03 to 0.03)	0.01 (−0.03 to 0.04)	−0.01 (−0.04 to 0.02)	−0.01 (−0.04 to 0.02)
P for trend	0.79	0.75	0.42	0.44
FEF_25-75_ (n=4086)				
Per SD increase	−0.02 (−0.05 to 0.01)	−0.02 (−0.06 to 0.01)	−0.02 (−0.05 to 0.01)	−0.02 (−0.06 to 0.01)
P for trend	0.19	0.13	0.27	0.13

*OR for asthma and atopy; difference in age, height and gender-adjusted SD units (β) for FEV_1_, FVC and FEF_25-75._

†Adjusted for iron supplementation during pregnancy and population substructure.

FEF_25-75_, maximal mid-expiratory flow; FEV_1_, forced expiratory volume in 1 s; FVC, forced vital capacity.

**Table 5:** Associations between maternal genotypic scores and atopy, asthma, FEV_1_, FVC and FEF_25-75_ in the offspring of women without iron supplementation in late pregnancy

	OR or β*† (95% CI)			
	Iron score	Ferritin score	Transferrin score	Transferrin saturation score
Atopy (n=2208)				
Per SD increase	1.08 (0.97 to 1.19)	1.06 (0.95 to 1.17)	0.97 (0.88 to 1.07)	1.05 (0.95 to 1.17)
P for trend	0.15	0.28	0.56	0.31
Asthma (n=2787)				
Per SD increase	1.09 (0.97 to 1.22)	0.98 (0.88 to 1.10)	1.00 (0.90 to 1.12)	1.08 (0.96 to 1.21)
P for trend	0.15	0.75	0.95	0.20
FEV_1_ (n=2300)				
Per SD increase	−0.04 (−0.08 to 0.00)	−0.02 (−0.06 to 0.02)	−0.03 (−0.07 to 0.01)	−0.05 (−0.09 to −0.01)
P for trend	0.07	0.38	0.13	0.03
FVC (n=2336)				
Per SD increase	−0.03 (−0.07 to 0.01)	−0.01 (−0.05 to 0.03)	−0.03 (−0.07 to 0.01)	−0.04 (−0.08 to 0.00)
P for trend	0.12	0.72	0.14	0.04
FEF_25-75_ (n=2336)				
Per SD increase	−0.02 (−0.06 to 0.02)	−0.02 (−0.06 to 0.02)	−0.02 (−0.06 to 0.02)	−0.02 (−0.06 to 0.02)
P for trend	0.35	0.39	0.36	0.28

*OR for asthma and atopy; difference in age, height and gender-adjusted SD units (β) for FEV_1_, FVC and FEF_25-75._

†Adjusted for population substructure.

FEF_25-75_, maximal mid-expiratory flow; FEV_1_, forced expiratory volume in 1 s; FVC, forced vital capacity.

Overall, there was no evidence for associations between the genotypic scores and childhood lung function (table 4); however, when restricted to unsupplemented mothers, negative associations were found between the transferrin saturation score and childhood FEV_1_ and FVC. There was also weak evidence for negative associations between the iron and transferrin scores and childhood FEV_1_ and FVC (table 5). No association with any outcome was observed among supplemented women (data not shown).

As a post hoc analysis, the individual SNP associations with the four iron biomarkers (based on GWA data^24^) were plotted against the individual maternal SNP associations with childhood FEV_1_ (online supplementary figure 2) and childhood FVC (online supplementary figure 3). These plots suggested that the weak associations between the maternal genotypic scores and FEV_1_ and FVC in the offspring of unsupplemented women were partly driven by rs1800562 (nearest gene: *HFE*). The simulation study conducted to assess the impact of collider bias on our results showed that, while collider bias did lead to inflated type 1 error rates, false positive rates were only substantially greater than nominal levels when the effect of iron levels on supplementation was extreme. For realistic values of this parameter, inflation of type 1 error rates was not substantial (see online supplementary file). When we studied the associations between the child’s genotypic scores and child’s outcomes, no association was found with childhood FEV_1_ or FVC. However, positive associations were found between the child’s iron and transferrin saturation scores and childhood atopy, and between the child’s ferritin score and childhood FEF_25-75_ (see online supplementary table 5). When we conducted MR-Egger and weighted median analyses to assess the associations between the maternal transferrin saturation score and childhood FEV_1_ and FVC among unsupplemented mothers, similar point estimates to the ones obtained using standard inverse variance weighted approach were found, suggesting no evidence of pleiotropy (online supplementary figures 4 and 5 for FEV_1_ and FVC, respectively). Weighted median analysis also suggested evidence for a statistically significant slope (p=0.05 and 0.03 for FEV_1_ and FVC, respectively).

## Discussion

Using an MR approach, we found weak evidence that a lower maternal iron status during pregnancy was associated with lower FEV_1_ and FVC in the offspring. These associations were only apparent in the absence of iron supplementation in late pregnancy. These results are in keeping with recent findings in ALSPAC in which we have found that lower maternal haemoglobin concentrations and anaemia in later pregnancy were associated with lower childhood lung function.^12^ They are also consistent with limited evidence from the SEATON study, suggesting that low prenatal iron status was associated with lower FEV_1_, although in that study the association was with ferritin in early pregnancy.^10^ In our study, statistical power was greater for analysis of lung function, a continuous outcome, than for analyses of binary outcomes.

A plausible mechanism for the associations we observed between the maternal genotypic scores and childhood lung function, and especially FEV_1_, could be that prenatal iron status influences growth and development of fetal lungs, especially airways. In support of this hypothesis, animal experiments have suggested that an adequate supply of iron is needed for optimal airway development. Chelation of iron by desferrioxamine in ex vivo lung buds from mouse embryos reduced the vascular network surrounding the developing lung buds and reduced epithelial branching; these inhibitory effects on vascular growth and epithelial branching were reversed by administering iron.^32^

### Strengths and limitations

One major strength of the ALSPAC birth cohort, apart from its size, population-based prospective design, rich information on numerous lifestyle factors and detailed phenotypic outcome measurements, is that maternal DNA was collected, enabling maternal genotyping and an MR approach; many birth cohort studies have not collected maternal DNA. To date, few studies have used an MR approach to investigate the role of prenatal nutrition in the aetiology of respiratory and atopic disorders in childhood, although two such studies have been conducted in ALSPAC.^33 34^ A limitation of using MR in our study is that, despite ALSPAC’s size, analyses may have been underpowered to detect small or modest effects, although we used genotypic scores to increase statistical power.^26^ The SNPs used to derive the maternal genotypic scores were selected from the largest GWA meta-analysis conducted so far on iron and iron biomarkers.^24^ Although the size and power of this GWA meta-analysis ensure that those SNPs are valid predictors of iron status in the general population, we cannot be sure that they are valid predictors of maternal iron status in pregnancy. Another limitation is that no iron biomarker had been measured in ALSPAC mothers during pregnancy to confirm directly that the genetic risk scores were valid IVs. Nevertheless, using maternal haemoglobin as a proxy for prenatal iron status, we showed that three key SNPs, and all four maternal genotypic scores, were strongly associated with haemoglobin concentrations in ALSPAC. We believe that this provides partial ‘internal validation’ of these genetic instruments as predictors of maternal iron status in pregnancy in the ALSPAC population. The fact that maternal genotypic scores were strongly associated with iron supplementation in late pregnancy, in a dose–response manner, provides further validation (those with the highest scores being most likely to develop iron deficiency anaemia, and hence to be given iron supplements, especially in late pregnancy when fetal demands are higher and anaemia is more common). It is worth emphasising that, despite supplementation, we still saw associations between all genetic instruments and haemoglobin concentrations among women. However, those associations were stronger among unsupplemented women, which is not surprising, as we would expect stronger genetic associations with iron biomarkers in the absence of iron supplementation. The fact that we also observed stronger associations between genotypic scores and lung function in children of unsupplemented mothers would support a causal interpretation. A major strength of using an MR approach is that the associations between the genetic instruments and outcomes should not be confounded. We confirmed that (with the exception of iron supplementation, occurring as a consequence of iron deficiency) the genotypic scores were not associated with a wide range of maternal and offspring characteristics, suggesting that our findings for lung function were unlikely to be confounded by lifestyle factors. The use of MR in the specific situation of testing maternal intrauterine effects on postnatal offspring outcomes, when there might be an overlap between maternal and offspring genetic variants, may lead to the violation of the exclusion restriction assumption of IV analyses. Several approaches have been proposed to address this issue.^31^ When we studied the associations between the child’s genotypic scores and childhood outcomes, no association was found with childhood FEV_1_ or FVC, suggesting that the associations that were found between the maternal genotypic scores and childhood FEV_1_ or FVC among unsupplemented mothers were not confounded by the child’s genetic variants. When we used MR-Egger and weighted median analyses, no evidence of pleiotropy was found and results supported a causal interpretation of the associations found between the maternal transferrin saturation score and childhood FEV_1_ and FVC among unsupplemented mothers. Nevertheless, we cannot totally rule out the possibility that there may be some residual pleiotropy and that the genetic associations might be confounded. Restriction of our analyses to white mothers, and adjustment for genetic markers derived by PCA, ensured that our findings were unlikely to be confounded by population substructure. Finally, the simulation study showed that it is unlikely that the results of this paper were driven by collider bias alone.

While there are multiple blood biomarkers of maternal iron status aside from iron, such as plasma ferritin, transferrin (iron is distributed systemically in the circulation as transferrin), transferrin saturation, or serum soluble transferrin receptor and its ratio to ferritin, no single marker of iron metabolism is considered ideal for assessment of iron deficiency, as each has limitations in terms of sensitivity and specificity.^35^ For example, serum iron is an unreliable indicator of availability of iron to the tissues because of wide fluctuation in levels due to recent ingestion of Fe, diurnal rhythm and other factors, such as infection.^36^ Transferrin saturation (ie, serum iron/total iron binding capacity×100%) is a more sensitive and specific indicator of iron deficiency than serum iron alone.^35^ However, it also fluctuates due to a diurnal variation in serum iron and is affected by the nutritional status. Serum ferritin is a stable glycoprotein that accurately reflects iron stores in the absence of inflammatory change and it is the first laboratory test to become abnormal as iron stores decrease and it is not affected by recent iron ingestion.^36^ However, serum ferritin is thought to be of limited usefulness in pregnancy because concentrations fall late in pregnancy, even when bone marrow iron is present^37^; this might partly explain why no association was seen between the genotypic ferritin score and lung function in our study. Another limitation of ferritin is that it is an acute phase reactant and concentrations will rise when there is active infection or inflammation.^38^ We therefore decided to derive four genotypic scores, predictive of four different iron biomarkers, to be able to assess prenatal iron status as comprehensively as possible. However, we cannot exclude the possibility that our findings for FEV_1_ and FVC occurred by chance, given the p values and the multiple analyses carried out; hence they should be interpreted with caution. Given the a priori nature of the hypothesis being tested, and the fact that some outcomes of interest are highly correlated, it did not seem appropriate to correct for multiple testing.

## Conclusions

Using an MR approach we have found weak evidence suggesting that low maternal iron status during pregnancy may cause impaired childhood lung function. There is need for further studies to strengthen causal inference. One way could be to conduct larger MR studies across multiple cohorts (if maternal genotype data are available), thus increasing statistical power. Another way could be to follow-up the offspring of mothers who have taken part in previous trials of iron supplementation in pregnancy, and to measure their lung function.^39^
