# Supplementary material for: Maternal iron status during pregnancy and respiratory and atopic outcomes in the offspring: a Mendelian randomisation study
Source: BMJ Open Respir Res. 2018 Mar 30;5(1):e000275. doi: 10.1136/bmjresp-2018-000275 (PMC5890059; doi:10.1136/bmjresp-2018-000275)
Supplement: Supplementary file 1 [file bmjresp-2018-000275supp001.pdf]

Online supplement document

Maternal iron status during pregnancy and respiratory and atopic outcomes in the offspring: a Mendelian randomization study

Annabelle Bédard[1], PhD, Sarah J Lewis[2], PhD, Stephen Burgess[3,4], PhD, A John Henderson[2*], MD, Seif O Shaheen[1*], PhD

[1]Centre for Primary Care and Public Health, Barts and The London School of Medicine and Dentistry, London, Queen Mary University of London, UK

[2] Population Health Sciences, Bristol Medical School, University of Bristol, UK

[3]MRC Biostatistics Unit, University of Cambridge, UK

[4]Cardiovascular Epidemiology Unit, University of Cambridge, UK

[*] Joint senior authors

Genotyping methods including quality control measures and imputation

Maternal genotyping

ALSPAC mothers were genotyped using the Illumina human660W-quad array at Centre National de Génotypage (CNG) and genotypes were called with Illumina GenomeStudio. PLINK (v1.07) was used to carry out quality control measures on an initial set of 10,015 subjects and 557,124 directly genotyped SNPs. SNPs were removed if they displayed more than 5% missingness or a Hardy-Weinberg equilibrium P value of less than 1.0e-06. Additionally SNPs with a minor allele frequency of less than 1% were removed. Samples were excluded if they displayed more than 5% missingness, had indeterminate X chromosome heterozygosity or extreme autosomal heterozygosity. Samples showing evidence of population stratification were identified by multidimensional scaling of genome-wide identity by state pairwise distances using the four HapMap populations as a reference, and then excluded. Cryptic relatedness was assessed using a IBD estimate of more than 0.125 which is expected to correspond to roughly 12.5% alleles shared IBD or a relatedness at the first cousin level. Related subjects that passed all other quality control thresholds were retained during subsequent phasing and imputation. 9,048 subjects and 526,688 SNPs passed these quality control filters.

We combined 477,482 SNP genotypes in common between the sample of mothers and sample of children. We removed SNPs with genotype missingness above 1% due to poor quality (11,396 SNPs removed) and removed a further 321 subjects due to potential ID mismatches. This resulted in a dataset of 17,842 subjects containing 6,305 duos and 465,740 SNPs (112 were removed during liftover and 234 were out of HWE after combination). We estimated haplotypes using ShapeIT (v2.r644) which utilises relatedness during phasing. We obtained a phased version of the 1000 genomes reference panel (Phase 1, Version 3) from the Impute2 reference data repository (phased using ShapeIt v2.r644, haplotype release date Dec

2013). Imputation of the target data was performed using Impute V2.2.2 against the reference panel (all polymorphic SNPs excluding singletons), using all 2186 reference haplotypes (including non-Europeans).

This gave 8,237 eligible children and 8,196 eligible mothers with available genotype data after exclusion of related subjects using cryptic relatedness measures described previously.

Child genotyping

DNA samples were extracted from lymphoblastoid cell lines, cord blood, or venous blood collected at 7 years of age, with a small number extracted from venous blood collected at 43-61 months. ALSPAC children were genotyped using the Illumina HumanHap550 quad chip genotyping platforms by 23andme subcontracting the Wellcome Trust Sanger Institute, Cambridge, UK and the Laboratory Corporation of America, Burlington, NC, US. The resulting raw genome-wide data were subjected to standard quality control methods. Individuals were excluded on the basis of gender mismatches; minimal or excessive heterozygosity; disproportionate levels of individual missingness (>3%) and insufficient sample replication (IBD < 0.8). Population stratification was assessed by multidimensional scaling analysis and compared with Hapmap II (release 22) European descent (CEU), Han Chinese, Japanese and Yoruba reference populations; all individuals with non-European ancestry were removed. SNPs with a minor allele frequency of < 1%, a call rate of < 95% or evidence for violations of Hardy-Weinberg equilibrium (P < 5E-7) were removed. Cryptic relatedness was measured as proportion of identity by descent (IBD > 0.1). Related subjects that passed all other quality control thresholds were retained during subsequent phasing and imputation. 9,115 subjects and 500,527 SNPs passed these quality control filters.

Simulation study

To investigate the impact of moderate to severe collider bias in the Mendelian randomization analyses stratified on iron supplementation status, we performed a simulation study. We simulated data on a genetic risk score, an exposure (haemoglobin as proxy for iron in our example), a confounder, supplementation status, and a continuous outcome variable for 6002 individuals, the same number as in the substantive analysis of this paper. For each individual indexed by i, the genetic risk score (g_i) was simulated as a normally distributed random variable, as was the confounder (u_i, assumed unmeasured), and the independent error terms for the exposure and outcome (ε_{Xi} and ε_{Yi}). The value of the exposure (x_i, representing an individual's pre-supplementation exposure) was calculated as a function of the genetic risk score, confounder, and an independent error term. We simulated whether that individual took iron supplementation (s_i) as a binomial random variable for which the probability of supplementation π_i depended on the values of the exposure and confounder. We also simulated an outcome variable (y_i) depending on the confounder and an error term. As the outcome did not depend on the exposure, the true causal effect of the exposure on the outcome was simulated as being null. This enabled us to investigate Type 1 error rates of the unstratified and stratified Mendelian randomization analyses.

The data-generating model was as follows:

$$x_i = \sqrt{0.01}\, g_i + 0.6\, u_i + \sqrt{1 - 0.01 - 0.36}\, \varepsilon_{Xi}$$

$$logit(\pi_i) = \ -0.4 \ + \ \beta_1\, x_i + \ u_i$$

$$s_i \sim Binomial(1, \pi_i)$$

$$y_i = \ 0.6\, u_i + 0.8\, \varepsilon_{Xi}$$

$$g_i, u_i, \varepsilon_{Xi}, \varepsilon_{Yi} \sim Normal(0, 1) \text{ independently}$$

The probability of supplementation was modelled as a function of the exposure. This leads to the collider (selection) bias in the stratified analyses: supplementation is a causal descendent from both the genetic variant and the confounder as it is downstream of the exposure, and the genetic variant and the confounder are determinants of the exposure. This leads to the genetic variant and confounder becoming correlated when the population is stratified on supplementation status, even though marginally the two variables are uncorrelated in the population as a whole(1). We note that collider bias would still occur if the probability of supplementation were a function of the exposure only, and would not occur if the probability of supplementation were a function of the confounder only.

Parameters were chosen so that the variances of the exposure and outcome were 1, the genetic score explained 1% of the variance in the exposure (i.e. $R^2 = 0.01$, similar to the substantive analysis of the paper), the prevalence of iron supplementation in the population was around 41%, and the odds ratio for supplementation per standard deviation increase in the exposure was $\exp(\beta_1)$. Confounding means that an observational association between the exposure and outcome is observed even though the causal effect of the exposure on the outcome is null.

We generated 10 000 simulated datasets in each of 7 scenarios with different values of β_1: [-2, -1, -0.5, 0, 0.5, 1, 2]. We assessed the association between the genetic variant and the outcome using linear regression in the whole population, and in the two strata (supplementation and no supplementation). Testing for an association between the genetic variant and the outcome is a valid test of the causal null hypothesis(2).

Table A shows the empirical Type 1 error rate at an alpha-level of 0.05 (the proportion of simulated datasets reporting a p-value less than 0.05) for association tests in the whole population, and in the strata of individuals with and without supplementation at each value of

β_1. Type 1 error rates for the unstratified analysis were close to nominal levels throughout. Similarly, Type 1 error rates were close to nominal levels when the selection parameter β_1 was close to zero. When the effect of the exposure on supplementation probability was large, some Type 1 error rate inflation was observed. Inflation was more marked when the effect of the exposure on supplementation probability was positive. However, a value of $\beta_1 = 2$ corresponds to an odds ratio for supplementation of $\exp(2) = 7.39$ per 1 standard deviation increase in the exposure. More realistic values of $\beta_1 = 0.5$, corresponding to an odds ratio of $\exp(0.5) = 1.65$ per 1 standard deviation increase in the exposure, led to very slight increases in Type 1 error rates from the nominal value of 5% up to around 6%. This suggests that collider bias is unlikely to explain the associations of the genetic variants with the outcome measures in the substantive analyses in this paper.

Hence, while we would be cautious not to generalize the result of this limited simulation study to other analysis contexts, in this case there seemed to be little potential for bias and Type 1 error rate inflation to arise due to collider bias.

Table A: Empirical power of association tests at 5% significance level in simulation study for different values of the selection parameter

Selection parameter β_1	effect $(\exp(\beta_1))$	Empirical power of association test in population (%)	Empirical power in supplementation stratum (%)	Empirical power in non-supplementation stratum (%)
-2	(0.14)	5.0	5.1	5.7
-1	(0.37)	5.2	5.6	5.8
-0.5	(0.61)	5.5	6.1	5.7
0	(1.00)	5.0	5.2	5.4
0.5	(1.65)	5.3	5.8	6.1
1	(2.72)	5.0	8.8	8.5
2	(7.39)	4.9	15.2	14.8

Online Table 1. Hardy-Weinberg equilibrium test for mothers' genotype

SNPs	Major/minor allele	P-value	N
rs1799945	C/G	0.61	6,130
rs1800562	G/A	0.72	6,137
rs855791	G/A	0.02	6,137
rs8177240	T/G	0.31	6,114
rs7385804	A/C	0.19	6,174
rs744653	T/C	0.31	6,137
rs651007	C/T	0.27	5,945
rs411988	A/G	0.29	6,137
rs9990333	C/T	0.04	6,137
rs4921915	A/G	0.51	6,125
rs6486121	T/C	0.38	6,137
rs174577	C/A	0.44	6,126

Online Table 2. Characteristics of mothers and offspring who had information on at least one of childhood outcome by weighted maternal genotypic iron score quartiles (n=6,002)

**Mother and offspring characteristics**	**Weighted iron score**				
	**Q1**	**Q2**	**Q3**	**Q4**	***P****
**Mother's age,** m (sd)	29.0 (4.6)	28.9 (4.6)	29.1 (4.6)	28.9 (4.3)	0.71
**Parity**, %					
0	47.9	48.3	42.9	47.2	
1	35.2	34.2	37.1	35.7	0.09
≥2	16.9	17.5	20.0	17.0	
**Sex of child**, %					
Male	49.2	50.6	49.4	50.5	0.80
Female	50.8	49.4	50.6	49.5	
**Season of birth**, %					
Winter	17.6	17.8	17.5	17.6	
Spring	24.7	25.6	25.7	23.3	0.69
Summer	30.8	29.5	27.9	31.0	
Autumn	27.0	27.2	29.0	28.2	
**Mother's educational level**, %					
Certificate of Secondary Education	13.7	13.3	13.1	13.9	
Vocational	9.2	8.0	9.2	8.8	0.13
Ordinary level	32.8	37.1	36.6	36.6	
Advanced level	27.5	24.5	27.5	26.1	
Degree	16.8	17.1	13.7	14.6	
**Housing tenure**, %					
Owned/mortgaged	85.9	84.2	85.5	85.3	
Council rented	8.0	8.8	9.1	8.6	0.67
Non-council rented	6.1	7.0	5.4	6.1	

**Financial difficulties**, %					
Yes	17.3	15.9	15.6	17.2	0.50
**Maternal history of atopic diseases,** %					
Yes	69.6	69.2	66.3	68.1	0.30
**Maternal anxiety score in pregnancy**, %	19.4	22.0	22.2	23.0	
0-9	28.3	25.4	27.0	26.5	0.48
10-14	25.9	25.7	25.6	25.3	
15-20	26.4	26.9	25.3	25.2	
≥20					
**Maximum maternal tobacco exposure**, %					
None	26.3	26.0	28.2	28.1	
Passive only	45.5	47.5	45.2	46.1	0.61
1-9 cig/day	9.1	8.5	8.4	6.9	
10-19 cig/day	11.2	11.0	10.4	11.4	
20+ cig/day	8.0	7.1	7.8	7.5	
**Maternal paracetamol use during pregnancy**, %	62.1	61.6	61.6	61.6	0.99
Yes					
**Maternal antibiotic use during pregnancy**, %	16.1	14.3	15.4	16.4	0.39
Yes					
**Maternal infections in pregnancy**, %					
Yes	46.3	45.9	45.7	44.1	0.60
**Maternal iron supplement use during pregnancy**, %	41.7	43.2	46.7	47.9	0.001
**Maternal iron supplement use during early pregnancy**, %	17.6	18.1	19.7	20.3	0.17
**Maternal iron supplement use during late pregnancy**, %	38.7	39.8	42.2	44.7	0.003

**Total energy intake**, m (sd)	7234 (1860)	7328 (1948)	7328 (1950)	7201 (1990)	0.16
**Maternal pre-pregnancy BMI**, %					
<18.50 kg/m²	4.7	3.8	3.2	4.4	
18.50-24.99 kg/m²	75.8	74.9	76.0	75.4	0.80
25.00-29.99 kg/m²	14.4	16.0	15.4	15.2	
≥30.00 kg/m²	5.2	5.4	5.5	5.1	
**Birth weight**, %					
<2500 g	3.7	3.0	3.3	3.7	
2500-2999 g	12.8	13.8	13.0	12.7	
3000-3499 g	36.0	34.7	36.9	36.9	0.93
3500-3999 g	34.3	35.3	33.0	33.2	
≥4000 g	13.3	13.2	13.9	13.5	
**Gestational age**, m (sd)	39.6 (1.8)	39.6 (1.6)	39.6 (1.7)	39.5 (1.7)	0.97

* F-statistics were used for differences in continuous variables and chi-squared tests for differences in categorical variables

Online Table 3. Associations between maternal genotypic scores and wheeze, eczema, hay fever and total IgE in the offspring

	OR or GMR±* (95% CI)			
	**Iron score**	**Ferritin score**	**Transferrin score**	**Transferrin saturation score**
**Wheeze (n=4,927)**				
Per SD increase	0.98 (0.90, 1.08)	0.90 (0.83, 0.99)	0.95 (0.87, 1.04)	0.98 (0.89, 1.07)
P for trend	0.72	0.03	0.27	0.59
**Eczema (n=4,917)**				
Per SD increase	0.99 (0.92, 1.07)	1.01 (0.93,1.09)	1.01 (0.93, 1.08)	1.00 (0.92,1.08)
P for trend	0.86	0.85	0.89	0.94
**Hay fever (n=4,907)**				
Per SD increase	0.98 (0.89, 1.08)	0.91 (0.83, 1.00)	0.93 (0.84, 1.02)	0.95 (0.86, 1.04)
P for trend	0.64	0.05	0.13	0.25
**Total IgE (n=3,179)**				
Per SD increase	0.98 (0.92, 1.04)	0.94 (0.89, 1.00)	0.98 (0.93, 1.04)	0.97 (0.92, 1.03)
P for trend	0.48	0.06	0.54	0.35

± odds ratio (OR) for wheeze, eczema and hay fever, and geometric mean ratio (GMR) for total IgE

* Adjusted for iron supplementation during pregnancy and population substructure

Online Table 4. Associations between maternal genotypic scores and wheeze, eczema, hay fever and total IgE in the offspring of women without iron supplementation in late pregnancy

	OR or GMR±* (95% CI)			
	**Iron score**	**Ferritin score**	**Transferrin score**	**Transferrin saturation score**
**Wheeze (n=2,817)**				
Per SD increase	1.00 (0.89, 1.13)	0.91 (0.81, 1.03)	0.99 (0.88, 1.11)	1.02 (0.90, 1.15)
P for trend	0.94	0.14	0.87	0.78
**Eczema (n=2,812)**				
Per SD increase	1.01 (0.92, 1.12)	1.06 (0.96,1.18)	1.05 (0.95, 1.16)	1.04 (0.94,1.16)
P for trend	0.80	0.26	0.37	0.40
**Hay fever (n=2,806)**				
Per SD increase	0.95 (0.83, 1.08)	0.93 (0.82, 1.05)	0.90 (0.79, 1.02)	0.93 (0.82, 1.05)
P for trend	0.40	0.25	0.10	0.24
**Total IgE (n=1,781)**				
Per SD increase	0.96 (0.89, 1.04)	0.95 (0.88, 1.03)	1.01 (0.94, 1.10)	0.97 (0.90, 1.05)
P for trend	0.34	0.22	0.71	0.45

± odds ratio (OR) for wheeze, eczema and hay fever, and geometric mean ratio (GMR) for total IgE

* Adjusted for population substructure

Online Table 5. Associations between child's genotypic scores and atopy, asthma, FEV_1, FVC and FEF_{25-75} in the offspring

	OR or β ±* (95% CI)			
	**Iron score**	**Ferritin score**	**Transferrin score**	**Transferrin saturation score**
**Atopy (n=3,503)**				
Per SD increase	1.11 (1.02, 1.20)	1.07 (0.99, 1.16)	1.02 (0.94, 1.11)	1.09 (1.00, 1.19)
P for trend	0.02	0.11	0.59	0.04
**Asthma (n=4,044)**				
Per SD increase	1.01 (0.92, 1.11)	1.08 (0.98, 1.19)	1.08 (0.99, 1.19)	1.06 (0.96, 1.17)
P for trend	0.83	0.13	0.10	0.24
**FEV_1 (n=3,557)**				
Per SD increase	-0.01 (-0.04, 0.03)	-0.01 (-0.04, 0.02)	-0.01 (-0.04, 0.02)	-0.01 (-0.04, 0.03)
P for trend	0.76	0.63	0.61	0.65
**FVC (n=3,615)**				
Per SD increase	0.01 (-0.03, 0.04)	0.02 (-0.01, 0.05)	-0.01 (-0.04, 0.02)	0.00 (-0.03, 0.03)
P for trend	0.65	0.25	0.60	0.93
**FEF_{25-75} (n=3,615)**				
Per SD increase	-0.02 (-0.05, 0.01)	-0.05 (-0.08, -0.02)	-0.01 (-0.05, 0.0é)	-0.02 (-0.05, 0.01)
P for trend	0.24	0.004	0.46	0.21

± odds ratio (OR) for asthma and atopy; difference in age, height and gender adjusted standard deviation units (β) for FEV_1, FVC and FEF_{25-75}

* Adjusted for iron supplementation during pregnancy and population substructure

Online Figure 1. Participant flow

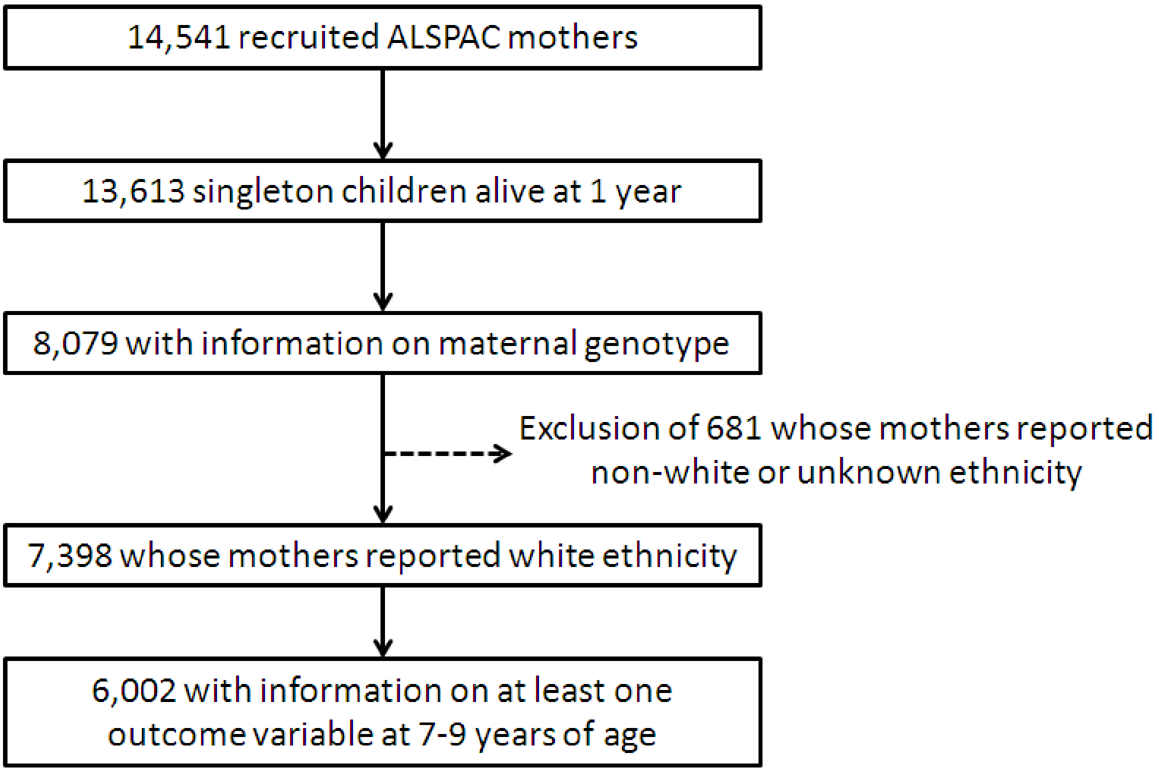

Online Figure 2. Genetic associations with iron biomarkers from GWA meta-analysis (horizontal axis) against genetic associations between maternal variants and FEV_1 in the offspring of women without iron supplementation in late pregnancy (vertical axis) (n=2,300)

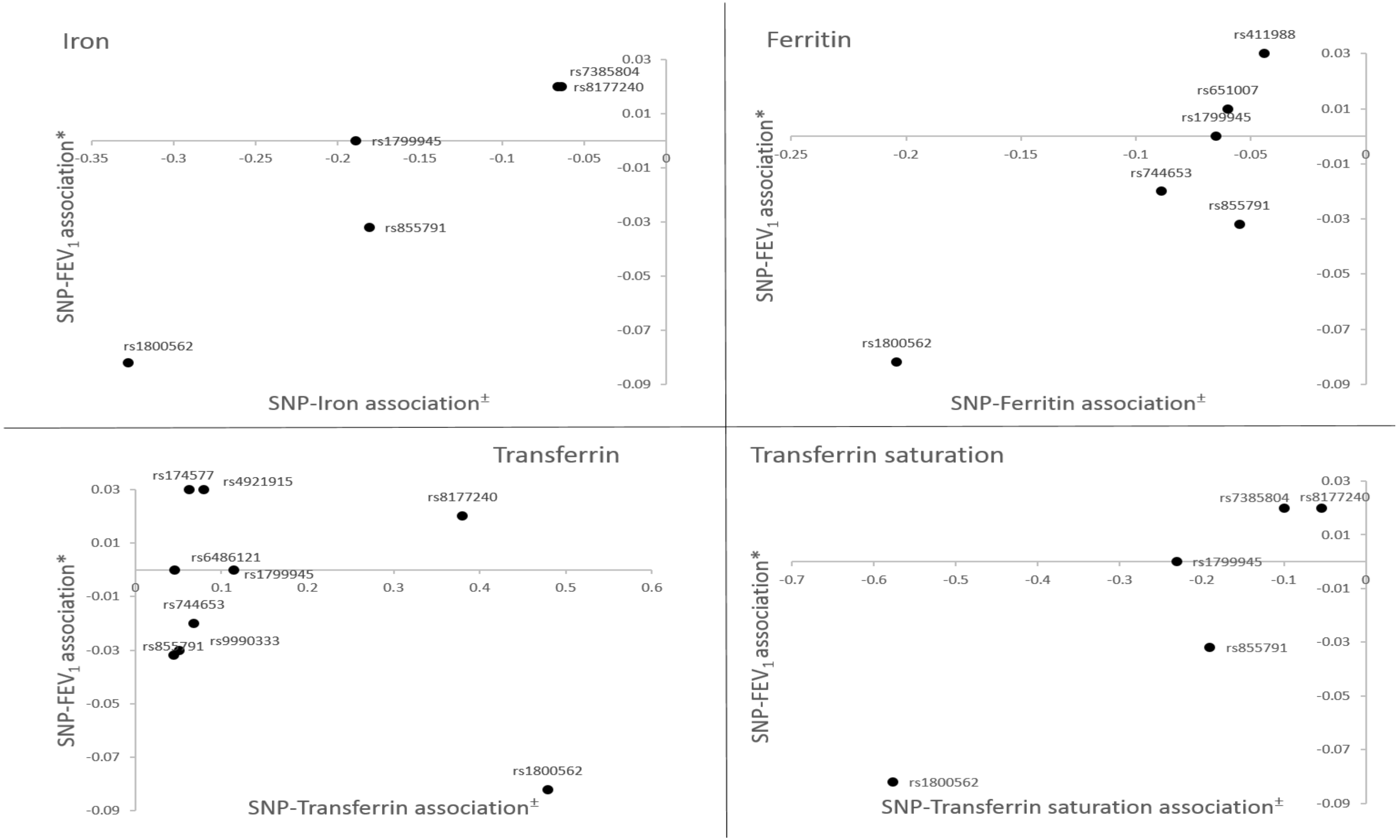

* per risk allele difference in age, height and gender adjusted standard deviation units (β) for FEV_1 for each SNP

± reported GWAS effect estimates for each SNP-iron biomarker (per risk allele) association (3)

NB: in the GWA meta-analysis by Benyamin et al. *Nat Commun* 2014 (3), estimates are reported for per risk allele (ie. allele associated with lower iron status, lower ferritin status, higher transferrin status or lower saturation status with $p < 5.10^{-8}$) effects

Online Figure 3. Genetic associations with iron biomarkers from GWA meta-analysis (horizontal axis) against genetic associations between maternal variants and FVC in the offspring of women without iron supplementation in late pregnancy (vertical axis) (n=2,336)

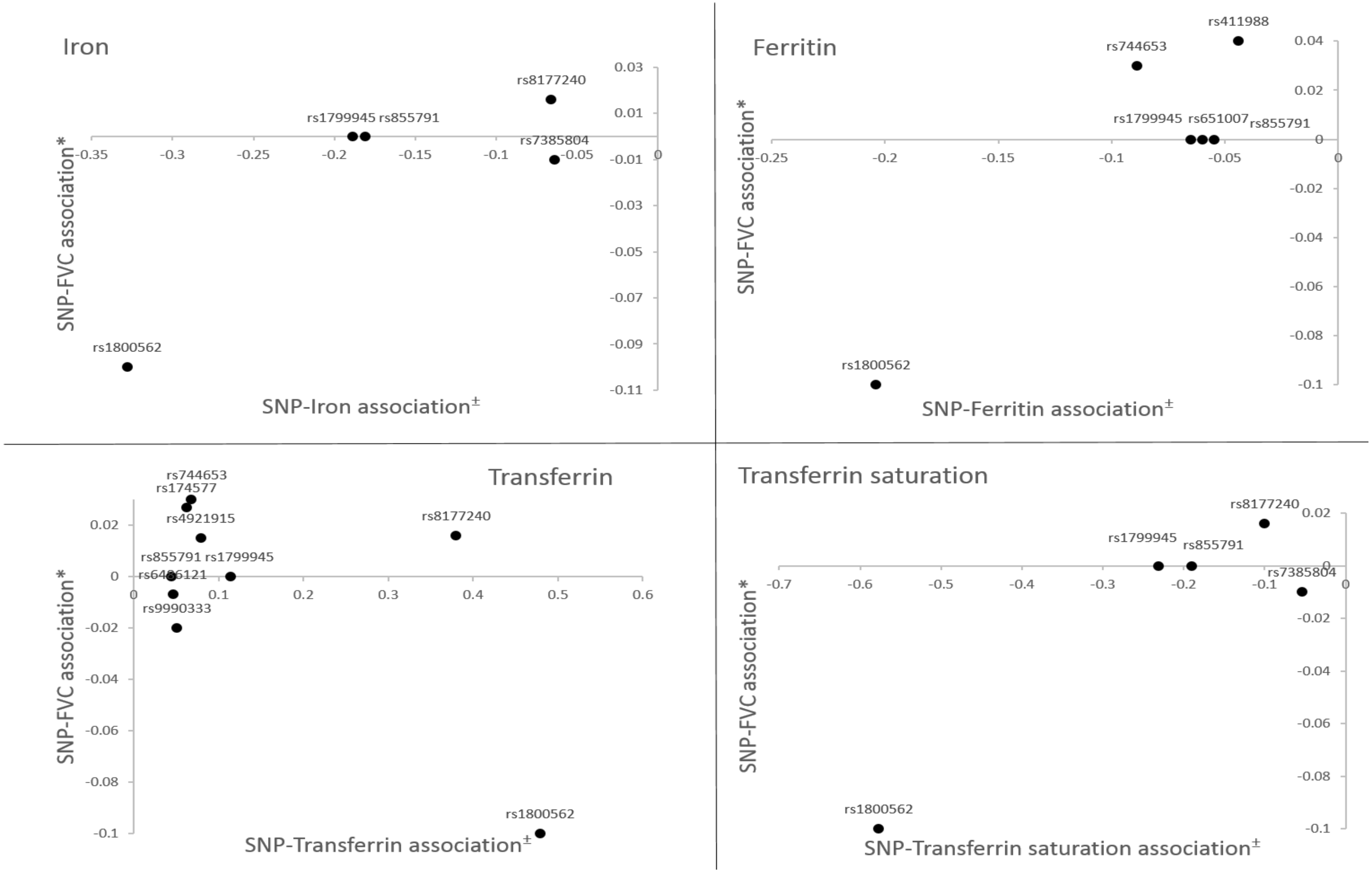

* per risk allele difference in age, height and gender adjusted standard deviation units (β) for FVC for each SNP

± reported GWAS effect estimates for each SNP-iron biomarker (serum iron, ferritin, transferrin, and transferrin saturation) association (3)

NB: in the GWA meta-analysis by Benyamin et al. *Nat Commun* 2014 (3), estimates are reported for per risk allele (ie. allele associated with lower iron status, lower ferritin status, higher transferrin status or lower saturation status with $p < 5.10^{-8}$) effects

Online Figure 4. Results of MR-Egger and weighted median analyses to assess the association between the maternal transferrin saturation score and childhood FEV_1 in the offspring of women without iron supplementation in late pregnancy

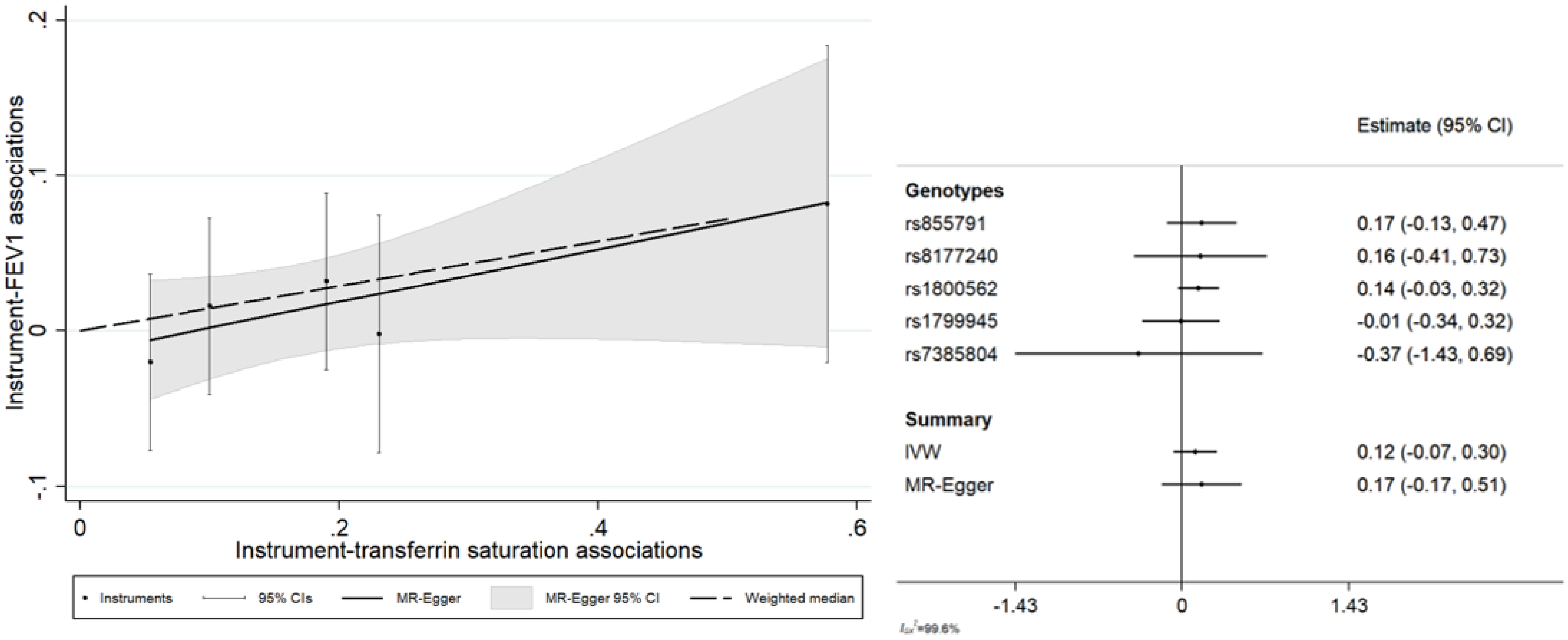

Online Figure 5. Results of MR-Egger and weighted median analyses to assess the association between the maternal transferrin saturation score and childhood FVC in the offspring of women without iron supplementation in late pregnancy

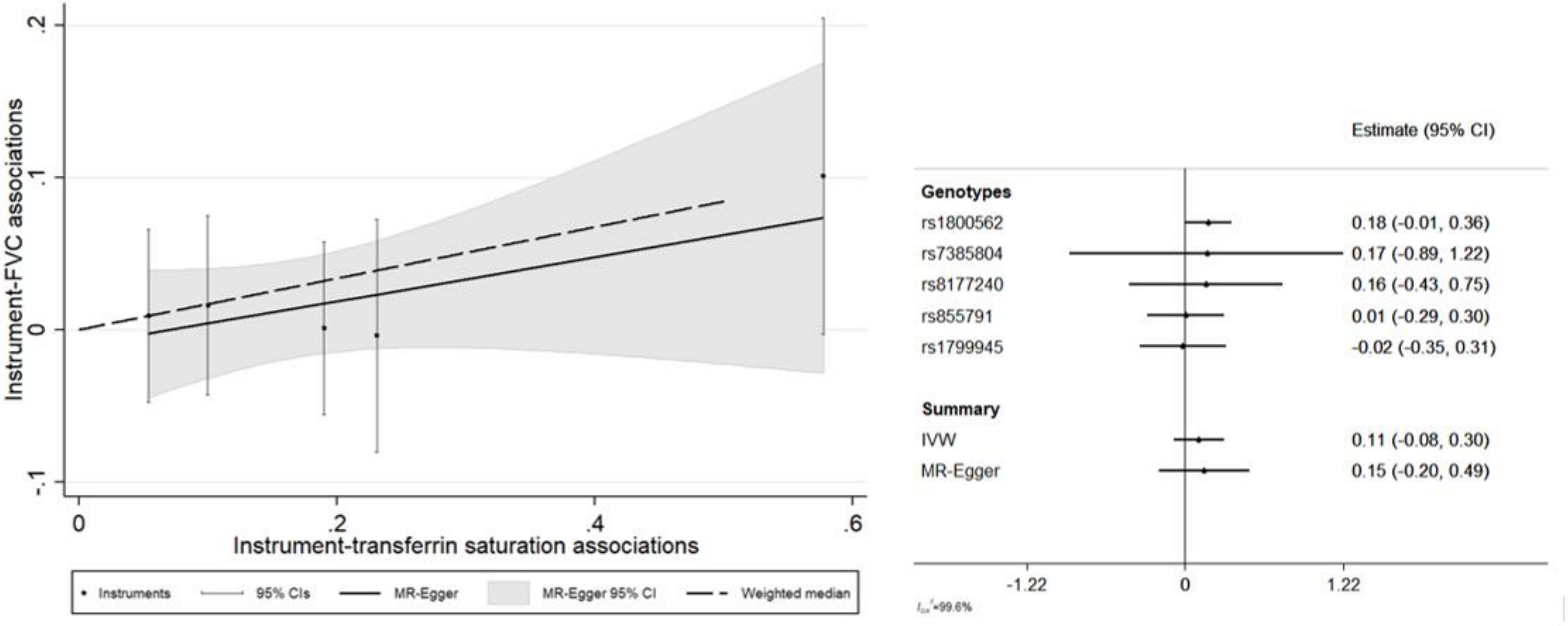

References

1. Cole SR, Platt RW, Schisterman EF, Chu H, Westreich D, Richardson D, Poole C. Illustrating bias due to conditioning on a collider. *Int J Epidemiol* 2010;39:417–420.

2. Didelez V, Sheehan N. Mendelian randomization as an instrumental variable approach to causal inference. *Stat Methods Med Res* 2007;16:309–330.

3. Benyamin B, Esko T, Ried JS, Radhakrishnan A, Vermeulen SH, Traglia M, Gögele M, Anderson D, Broer L, Podmore C, Luan J, Kutalik Z, Sanna S, van der Meer P, Tanaka T, Wang F, Westra H-J, Franke L, Mihailov E, Milani L, Hälldin J, Häldin J, Winkelmann J, Meitinger T, Thiery J, Peters A, Waldenberger M, Rendon A, Jolley J, *et al.* Novel loci affecting iron homeostasis and their effects in individuals at risk for hemochromatosis. *Nat Commun* 2014;5:4926.
